# Potential Impacts of Climate Change on Insect Communities: A Transplant Experiment

**DOI:** 10.1371/journal.pone.0085987

**Published:** 2014-01-22

**Authors:** Sabine S. Nooten, Nigel R. Andrew, Lesley Hughes

**Affiliations:** 1 Hawkesbury Institute for the Environment, University of Western Sydney, Penrith, New South Wales, Australia; 2 Centre for Behavioural and Physiological Ecology, Zoology, University of New England, Armidale, New South Wales, Australia; 3 Department of Biological Sciences, Macquarie University, Sydney, New South Wales, Australia; Clemson University, United States of America

## Abstract

Climate change will have profound impacts on the distribution, abundance and ecology of all species. We used a multi-species transplant experiment to investigate the potential effects of a warmer climate on insect community composition and structure. Eight native Australian plant species were transplanted into sites approximately 2.5°C (mean annual temperature) warmer than their native range. Subsequent insect colonisation was monitored for 12 months. We compared the insect communities on transplanted host plants at the warmer sites with control plants transplanted within the species' native range. Comparisons of the insect communities were also made among transplanted plants at warmer sites and congeneric plant species native to the warmer transplant area. We found that the morphospecies composition of the colonising Coleoptera and Hemiptera communities differed markedly between transplants at the control compared to the warmer sites. Community structure, as described by the distribution of feeding guilds, was also found to be different between the controls and transplants when the entire Coleoptera and Hemiptera community, including non-herbivore feeding guilds, was considered. However, the structure of the herbivorous insect community showed a higher level of consistency between plants at control and warm sites. There were marked differences in community composition and feeding guild structure, for both herbivores and non-herbivores, between transplants and congenerics at the warm sites. These results suggest that as the climate warms, considerable turnover in the composition of insect communities may occur, but insect herbivore communities may retain elements of their present-day structure.

## Introduction

The distribution, abundance, physiology, behaviour and ecology of all species will be affected by climate change [1]–[7]. Species are expected to respond idiosyncratically, resulting in changes in interactions, such as competition, predation or parasitism, with far-reaching consequences for community structure, composition and function [8]–[10]. The decoupling of present-day interactions between plants and insects may be particularly important. Insects have already responded to climatic changes over the past few decades, via range shifts and changes in phenology [11]–[16]. Mismatches in interactions between species have occurred, due to temporal [17], [18] and spatial [13]–[15] decoupling. Further, significant changes in the structure of species assemblages are already apparent in both temperate and tropical regions (e.g. [19]–[21]).

Increasing temperature may have particularly profound impacts on the composition of insect communities because it will affect almost all life history parameters, including emergence, growth rate, and voltinism [22], [23]. A field-based warming experiment that manipulated several factors (temperature, CO_2_ and water) showed that temperature had the largest effect on insect community composition and structure as a result of individualistic responses of both individual species and of different feeding guilds [24].

Disruptions of current plant-insect communities may have particularly far-reaching consequences for terrestrial ecosystems because plants and their associated phytophagous insects comprise a major proportion of terrestrial biodiversity - approximately 50% of all described species [25]–[27]. Insects perform many important ecosystem services, such as pollination, predation, and parasitism; they consume plant tissue (herbivory) [28], [29], but they can also be significant pests [30], [31].

### What shapes plant-insect communities?

Understanding the factors that currently shape plant-insect communities is fundamental to predicting how such assemblages will be affected as the climate continues to change. Several non-mutually exclusive factors have been suggested as important drivers affecting the composition and structure of plant-insect communities. For example, MacArthur [32] suggested that community assembly may be chiefly driven by climatic factors, and that these may operate via impacts on species interactions. In contrast, Strong et al. [26] suggested that the major drivers of the phytophagous community are the physical and chemical characteristics of the host plants. In the present study, we tested the roles of both the host plant and climatic factors as possible drivers of plant-insect community assembly, under current and warmer climate conditions.

### How can we predict climate change impacts on communities?

There are significant challenges for predicting future impacts of climate change at the community level and several approaches have been taken [7], [33]. Dynamic vegetation models have been developed to project future changes in plant communities (e.g. [34], [35]), field surveys have examined turnover of community composition and structure along either altitudinal (e.g. [36]–[38]) or latitudinal gradients (e.g. [39], [40]), and field-based warming experiments have generally investigated changes in plant-insect associations on a smaller scale (e.g. [24], [41]).

Transplant experiments offer powerful, though rarely used, tools for assessing potential impacts of climate change at the community level. Those transplant experiments that have been performed have mostly focused on plant [42]–[47] or soil communities [48]–[52], with only a few focused on plant-insect assemblages [53]–[55].

In this study we used a multi-species transplant experiment to investigate potential changes in plant-insect communities under a warmer climate. We compared community composition, in terms of the number and identity of morphospecies, and community structure, in terms of feeding guilds [56], [57] on plants at warmer sites, compared to those within their native range, and to those on closely-related host species native to the warmer sites.

## Materials and Methods

The field transplant experiment was conducted in eastern Australia. Eight plant species were grown from seed then planted into field sites (i) within the species' native range and (ii) outside the native range into a warmer climate. Subsequent insect colonisation was monitored for one year.

### Host plant species

Eight host plant species from three major Australian plant families were chosen, based on three criteria: all species were native to Australia, had a relatively narrow distribution within or close to the Sydney Basin, and all were locally common in dry sclerophyll forest habitats. From the Fabaceae (subfamily Mimosoideae): *Acacia parvipinnula* Tindale, (subgenus Phyllodineae), *A. obtusata* Sieber ex DC (subgenus Phyllodineae), and from subfamily Faboideae, *Daviesia corymbosa* Sm. From the Myrtaceae: *Angophora hispida* Sm. Blaxell, *Callistemon pinifolius* J.C. Wendl., and *Leptospermum squarrosum* Gaertn. From the Proteaceae: *Hakea gibbosa* Sm. Cav. and *Telopea speciosissima* Sm. R. Br. Each plant species has a fairly narrow distribution in coastal south-east New South Wales, including the Sydney Basin and extending latitudinally from approximately Newcastle (32° 55′ 33.6"S, 151° 46′ 51.6"E) in the north, to Nowra (34° 52′ 22.8"S, 150° 36′ 10.8"E) in the south. All the species are common understory shrubs in the vegetation type *Sydney Coastal Dry Sclerophyll Forest* [58], growing on low-nutrient, freely draining soils derived from Hawkesbury sandstone [59]. Collectively, the distributions of the species range from 0–700 m in elevation, with average precipitation of 1000–1300 mm p.a. [58] and approximately 17.7°C average annual temperature [60].

All plants were established from seed in the glasshouse facilities at Macquarie University in January 2009. Seeds from Fabaceae species were pre-treated with boiling water; no other seeds required any pre-treatment. Once germinated, seedlings were transferred into 5 cm square tubes filled with a potting – sand mix (4∶1 ratio), and slow release fertilizer was applied. Seedlings were subjected to the natural photoperiod and watered twice daily. When roots were established, plants were transferred into 13 cm pots and if necessary after six months, were transferred once more into 25 cm square pots to prevent root circling. After seven months, plants were placed outside the glasshouse to acclimatise to natural weather conditions, and grown for a further six months. One species, *Acacia parvipinnula*, had to be successively cut back to 150 cm because it grew more vigorously than the other species.

### Field sites

**Figure 1:**
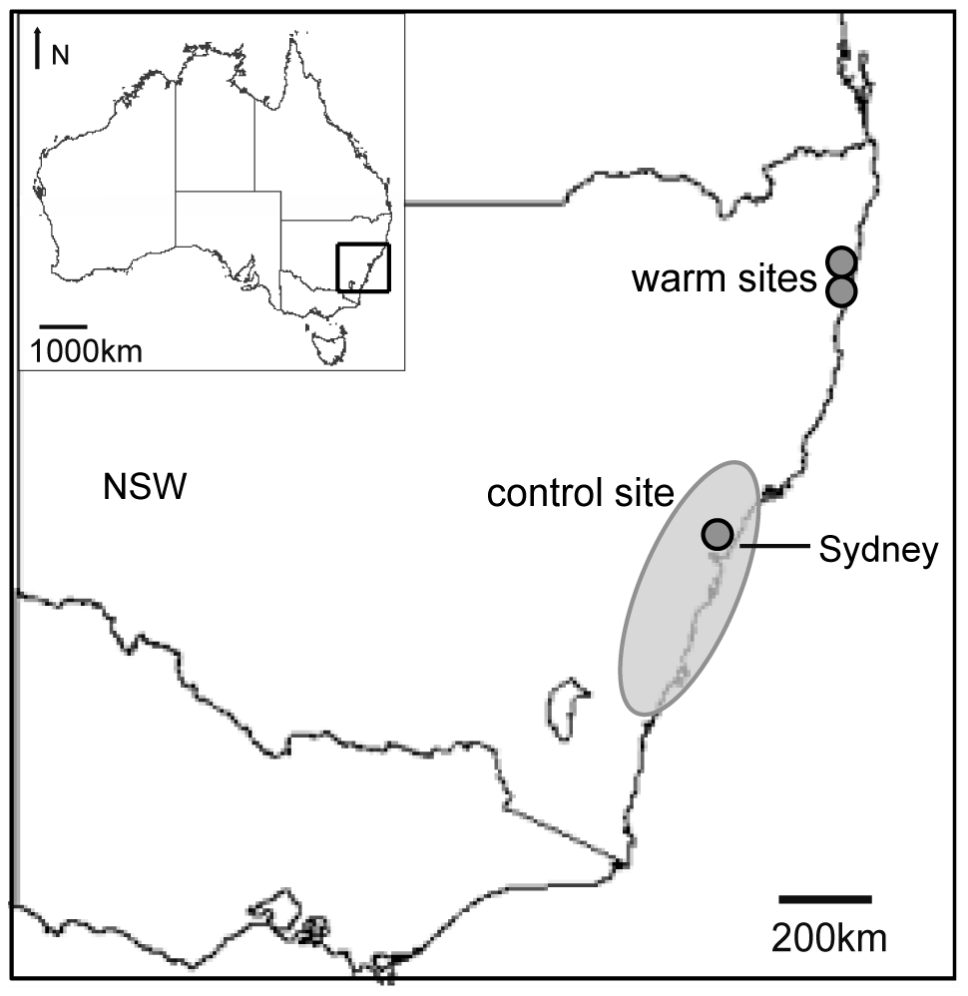
Location of three transplant sites. One control site within the current range of eight host plant species (grey oval); and two warm sites, located ca. 600 km north of the northern boundary of the plant species' range.

We selected three field transplant sites, one control site and two warmer sites. There was a temperature difference of approximately +2−3°C between control and warm sites, but all had similar precipitation patterns and soil type, and all were located in similar vegetation. One site was located in the approximate centre of all the plant species' native ranges, at Mt. Ku-ring-Gai (33° 39′ 39.3798"S, 151° 8′ 5.6472"E), 38 km north of Sydney, referred to hereafter as the control site (C). The two warmer sites were located near Grafton in northern New South Wales (NSW), ca. 600 km north of the northern-most boundaries of the species' native ranges. The sites were located 8 km apart, one in Minnie Water (29° 46′ 26.76"S, 153° 17′ 23.244"E) hereafter referred to as warm site 1 (W1) and the other in Wooli (29° 53′ 8.124"S, 153° 15′ 58.752"E) referred to as warm site 2 (W2) (Fig. 1).

Mean annual temperature, calculated over the last 30 years, near the control site was 17.25°C (nearest weather station: Sydney) and 19.75°C near the two warmer sites W1 and W2 (nearest weather station: Grafton) [60]. The mean annual precipitation at the warmer sites (1340 mm) was slightly higher than at the control site over the same period (1164 mm). The difference in mean annual temperature of approximately 2.5°C between the control and the two warmer sites reflects the current projections for warming in NSW by the year 2050 [61]–[63]. Annual precipitation for the three sites is within the projected patterns for mid- and north-coastal areas of NSW. The control site and W1 were situated in dry sclerophyll forest with 10–30% canopy cover of tall eucalypt trees, mainly *Angophora costata*. The W2 site was located in coastal heath, close to dry sclerophyll forest, with 5–15% canopy cover dominated by *Banksia* spp.

Soil types at the transplant sites were sandy and freely draining, similar to those at native sites of the plant species. Soil fertility, in terms of N, P and K, at all native and transplant sites was low. Six soil samples (10 cm diameter×10 cm depth) were taken at each transplant site and at two sites within Ku-ring-Gai Chase National Park (where most of the eight plant species naturally co-occur), with soils derived from the Hawkesbury sandstone. Soil samples were tested for total Nitrogen, Phosphorus and Potassium content by a commercial soil testing facility (SESL, Thornleigh, NSW, Australia). Total N, P and K values for the control site (N: 0.113% w/w, P: 0.005% w/w and K: 0.001%) were slightly higher than W1 (N: 0.053% w/w, P: 0.002% w/w and K: 0.012%) and W2 (N: 0.03% w/w, P: 0.007% w/w and K: 0.021%) but comparable to the two sites in Ku-ring-Gai Chase National Park (N: 0.078% w/w, P: 0.002% w/w and K: 0.046%).

All transplant sites were fenced to exclude vertebrate herbivores. Shortly before being transported to the transplant sites, all plants were sprayed with insecticide (0.6% pyrethrum/water solution) to remove all external insects that may have colonised the plants during their establishment at Macquarie University. In March/April 2010, 640 plants were transplanted into the three sites: 160 individual plants at the control site (20 plants per species) and 240 plants each at W1 and W2 (30 individual plants per plant species per site). The eight species were planted in random positions within each site, 1.5 m apart in a 36 m×12 m grid. Plants were watered in for three days after transplantation. The experiment was carried out for 12 months, from April 2010 to April 2011.

### **Insect Collection**

Arthropods were collected on three occasions, in September and December 2010, and in March 2011, with pyrethrum knockdown following the protocol of Andrew & Hughes [40]. Sampling occurred in the morning between 7.00 am and 11.00 am on low wind days. At each site, at each collection event, ten individual plants per host plant species were haphazardly selected and sprayed with a 0.6% pyrethrum/water solution. All arthropods that fell onto four collecting trays (each 50×30 cm) previously placed beneath the plant were transferred into 70% ethanol. Using the same protocol, collections were also made from four plant species native to the warm transplant area, belonging to the same genera as five of the transplanted species: *Acacia longifolia* Andrews, subsp. *sophorae* Labill. (Fabaceae, subgenus Phyllodineae), *Callistemon pachyphyllus* Cheel (Myrtaceae), *Hakea actites* W.R. Barker (Proteaceae) and *Leptospermum trinervium* Sm. Joy Thomps. (Myrtaceae). Ideally, insects would have been collected from congeneric plant species for all eight transplant species, but only four could be located in the warm transplant area.

We also investigated differences in plant growth and herbivory between control and warm sites. Firstly, the height of all individual plants at the time of transplantation and again after 12 months was measured. Secondly, total herbivory experienced by an individual plant after 12 months was estimated *in situ*: 10 individual plants per species and site were randomly selected. For each plant two branches were selected and each leaf was visually assessed for the percentage missing or damaged leaf area, and an average value for herbivory was then estimated.

### Insect community characterisation

We focused on the orders Coleoptera and Hemiptera because they were dominant within the samples. Insect identification followed the routine described in Nipperess et al. [64]. All adult insects from the Coleoptera and Hemiptera were sorted to morphospecies using the protocol from Oliver & Beattie [65], and subsequently identified to family level. We excluded larvae (Coleoptera) and nymphs (Hemiptera) from the analyses because of the difficulty relating them to the corresponding adults [53]. Adult Coleoptera and Hemiptera were assigned to functional feeding guilds, based on the morphology of their mouthparts, feeding method and targeted plant tissues. Feeding guilds were assigned to whole families by choosing the feeding type expressed by the largest number of members of the family following the descriptions by Lawrence & Britton [66], Andrew & Hughes [39], [40], [53] and Nipperess et al. [64]. An exception to this method was the family Cicadellidae (Hemiptera), in which morphospecies were identified to subfamily level for feeding guild assignments, using the identification key provided by Fletcher [67]; this was necessitated by the highly heterogeneous nature of feeding habits within this taxon. Morphospecies within the order Coleoptera were divided into four feeding guilds: leaf chewers, fungivores, predators and scavengers. Morphospecies within the order Hemiptera were assigned to one of five feeding guilds: mesophyll feeders, phloem feeders, xylem feeders, predators, and seed predators (Table S1 in file S1).

Two types of comparisons were made for the insects collected from each host plant species. Firstly, we compared numbers of morphospecies within feeding guilds from the entire collection of Coleoptera and Hemiptera (hereafter known as the ‘full dataset’). For the full dataset, we pooled the mesophyll, phloem and xylem feeders into a general guild of ‘sapsuckers’, as we were interested in general patterns within both herbivore and non-herbivore guilds. Secondly, we compared only phytophagous species within these orders, referred to hereafter as the ‘herbivore dataset’, using a more finely divided herbivore feeding guild structure.

### Statistical analyses

#### Host plant performance

We investigated possible site effects on plant height and leaf herbivory after 12 months using separate one-way ANOVAs (SPSS v20) for (i) all plant species pooled and (ii) for each host plant species separately. A single two-way ANOVA was not used because for individual plant species the assumption of homogeneity of variance (assessed by Levene's test), even after transformation, could not always be met. In these instances a Welch's ANOVA (resulting in fractional degrees of freedom), which is more robust to violation of this assumption, was used [68]. Tukey's post hoc tests were performed to test between means. Growth data were square root transformed to improve normality where appropriate. Herbivory data were logit transformed instead of the commonly used arcsine, following suggestions by Warton & Hui [69].

#### Coleoptera and Hemiptera community composition and structure

Data for each of the three collection events were pooled to produce as complete samples of the insect fauna on each host plant species as possible. For each plant species we compared the insect community in terms of (1) morphospecies composition and (2) the distribution of each feeding guild among sites. Comparisons were performed using both the full and the herbivore dataset. To assess differences in community composition among sites and congenerics, we firstly compared morphospecies overlap among sites within and among plant species using the SIMPER function in the programme PRIMER *v*6 [70]; we then compared morphospecies composition for pooled plant species using Bray-Curtis dissimilarity matrices, based on square root transformed morphospecies richness data, to generate non-metric multidimensional scaling (nMDS) plots.

Community structure, in terms of the number of morphospecies within feeding guilds, was compared using the multivariate extension of generalised linear models (mGLM), based on negative binomial regression [71]. The computation was conducted using the mvabund package [72] in R 2.14.1 [73]. We investigated whether there was consistency in guild structure among plant species within plant families at the control site. Then two hypotheses about possible drivers of guild structure were tested: firstly, that guild structure is chiefly associated with host plant identity [26], and secondly, that guild structure is chiefly associated with climatic factors [32]. To examine the role of host plant identity as a driver, we compared guild structure (i) between the control site and each warmer site W1 and W2, and (ii) between W1 and W2. To examine the role of climatic factors, we compared guild structure on five of the transplanted plant species (iii) between each warmer site W1 and W2 and the congeneric host plant species in the warmer transplant area.

## Results

### Host plant performance

Overall, growth for pooled plant species was not significantly different among sites (F_2,407_ = 2.169, p = 0.116). For individual plant species, growth was significantly different among sites for three of the eight plant species: *A. obtusata* grew more at W2, *C. pinifolius* grew less at W1 and *T. speciosissima* grew more at the control site (Table S2 in file S1). Overall, average leaf herbivory did not differ significantly among sites (Welch-F_2,153.5_ = 0.773, p = 0.463), although herbivory on *A. parvipinnula* was significantly higher at W1, (F_2,15.9_ = 5.715, p<0.013).

### Community composition

#### Comparison of transplanted plant species at control and warm sites

A total of 354 morphospecies of Coleoptera (n = 97) and Hemiptera (n = 257) were collected from the transplanted plants at the three sites. Of these 273 (77%) were classified as phytophagous (Coleoptera n = 30, Hemiptera n = 243). Overall, there was little commonality in morphospecies identity among the three sites (Table 1). There was little similarity in morphospecies among the host species within the three plant families; in the Fabaceae only 3.2% overlap of morphospecies was found at all three sites (full dataset, averaged across plant species) and even less overlap for the herbivore dataset (1.2%). In the Myrtaceae there was an average of 2.3% overlap in morphospecies among all three sites (full dataset), and only 0.6% for the herbivore dataset. In the Proteaceae there was an average of 3.1% overlap for the full dataset and 0.3% for the herbivore dataset (Table 1).

**Table 1:** Similarities (%) of Coleoptera and Hemiptera morphospecies from eight host plant species among transplant sites and congeneric plant species in the warm area, for (i) the full dataset and (ii) the herbivore subset.

Plant species	Similarities (%)				
	C-W1 a	C-W2	W1-W2	conge-W1	conge-W2
(i)					
Fabaceae average	1.6	0.8	7.3		
*A. obtusata*	1.2	1.3	6.4	1.6	1.2
*A. parvipinnula*	3.2	1.0	4.9	5.2	2.8
*D. corymbosa*	0.5	0.1	10.6		
Myrtaceae average	0.6	1.0	5.4		
*A. hispida*	1.1	2.1	4.2		
*C. pinifolius*	0.3	0.1	4.7	0.5	1.1
*L. squarrosum*	0.5	0.8	7.4	2.0	1.5
Proteaceae average	1.9	3.6	3.8		
*H. gibbosa*	2.8	5.8	3.9	0.2	0.5
*T. speciosissima*	1.1	1.3	3.7		
(ii)					
Fabaceae average	1.5	0.2	2.0		
*A. obtusata*	0.7	0.1	2.3	0.7	0.5
*A. parvipinnula*	3.0	0.6	3.6	5.7	3.0
*D. corymbosa*	0.8	0.0	0.0		
Myrtaceae average	0.6	0.4	0.7		
*A. hispida*	1.2	0.1	0.0		
*C. pinifolius*	0.5	0.0	1.7	0.1	0.0
*L. squarrosum*	0.0	0.3	0.4	2.0	0.1
Proteaceae average	0.3	0.4	0.4		
*H. gibbosa*	0.5	0.8	0.0	0.2	0.1
*T. speciosissima*	0.0	0.0	0.7		

a Sites: control (C), warm 1 (W1) and warm 2 (W2), congeneric plant species in the warm area (conge).

In the full dataset, the morphospecies similarity for individual host plant species among sites was low (Table 1). For the full dataset, values ranged from 0.1% (*C. pinifolius*) to 10.6% (*D. corymbosa*), for the herbivore dataset from 0% for four of the plant species to 3.6% (*A. parvipinnula*). As for the plant families, individual plant species supported more co-occurring morphospecies within the full dataset than for the herbivore dataset. For the full dataset, similarities were higher between the two warm sites W1 and W2 than between these sites and the control.

When morphospecies co-occurred at individual plant species among transplant sites, they tended to be fungivores from the families Ptiliidae (1 morphospecies) and Lathridiidae (2 morphospecies) (Coleoptera), phloem feeders from the families Aphididae (2 morphospecies) and Coccidae (2 morphospecies) (Hemiptera) and mesophyll feeders from the family Cicadellidae, subfamily Typhlocybinae (2 morphospecies) (Hemiptera) (Table S3 in file S1). Numbers of co-occurring morphospecies between the control and the warm sites ranged from 1 (*D. corymbosa*) to 10 (*A. parvipinnula*), and between W1 and W2 they ranged from 2 (*T. speciosissima*) to 23 (*A. parvipinnula*).

#### Comparison of congeneric and transplanted plant species at the warm sites

A total of 271 morphospecies of Coleoptera (n = 136) and Hemiptera (n = 135) were collected from 120 individual plants of the four congeneric plant species native to the warm transplant area. Of these, 66% were classified as herbivores (Coleoptera n = 152, Hemiptera n = 197). There were few morphospecies in common between the transplants at W1 and W2 and the native congeneric plant species (Table 1). For the full dataset, values ranged from 0.2% (*H. gibbosa* and congeneric *Hakea*) to 5.2% (*A. parvipinnula* and congener). For the herbivore dataset, values ranged from 0% (*C. pinifolius* and congener) to 5.7% (*A. parvipinnula* and congener). On average, there was slightly more overlap in morphospecies for the full dataset (1.7%) than for the herbivore dataset (1.2%).

Co-occurring morphospecies tended to be fungivores from the families Ptiliidae (1 morphospecies) and Lathridiidae (2 morphospecies) (Coleoptera), phloem feeders from the family Aphididae (2 morphospecies) (Hemiptera), predators from the families Cantharidae (1 morphospecies) and Staphylinidae (1 morphospecies) (Coleoptera) and scavengers from the family Scarabaeidae (1 morphospecies) (Coleoptera) (Table S3 in file S1). Numbers of co-occurring morphospecies between the congeneric plant species and any warm site ranged from 6 (*H. actites* and *H. gibbosa*) to 42 (*A. longifolia* and *A. parvipinnula*).

#### Comparisons among sites and congeneric plant species

There was little similarity in community composition for each of the eight transplanted plant species among the three transplant sites, and among transplants and their congeners, as indicated by non-metric multidimensional scaling (nMDS) plots (Figure S1-S3 in file S1). Community composition for morphospecies of both the full and herbivore dataset showed little clustering in relation to the transplant sites or congeneric plant species.

### Community structure

#### Comparison of transplanted plant species at control and two warm sites

**Figure 2:**
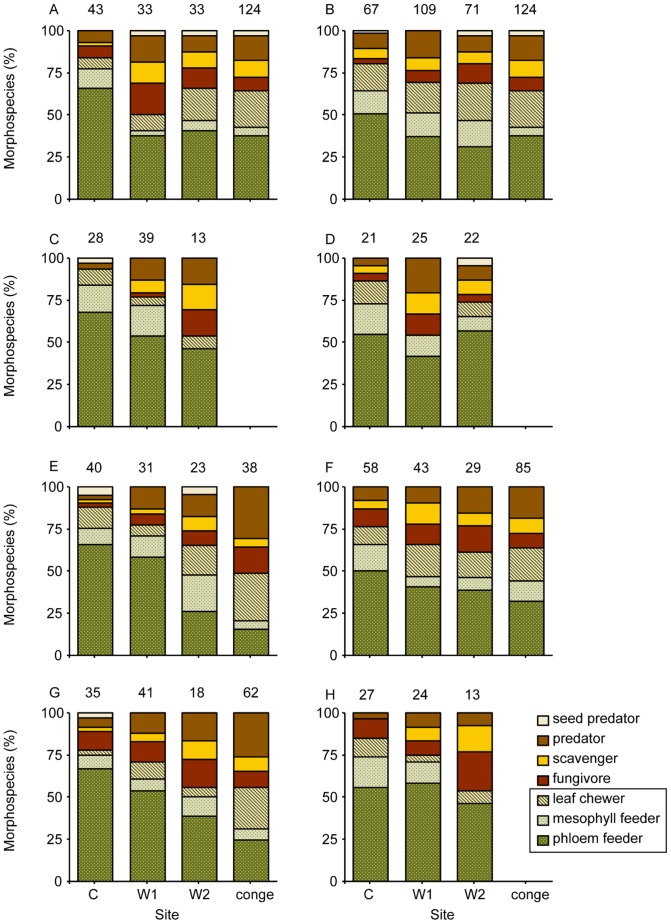
Coleoptera and Hemiptera feeding guild structure from eight plant species at three transplant sites. *A. obtusata* (A), *A. parvipinnula* (B), *D.corymbosa* (C), *A. hispida* (D), *C. pinifolius* (E), *L. squarrosum* (F), *H. gibbosa* (G) and *T.speciosissima* (H) at control (C), warm 1 (W1) and warm 2 (W2), and congeneric plant species (conge) at the warm sites. Herbivore feeding guilds are ‘hatched’; mesophyll and phloem feeders combined are ‘sapsuckers’; numbers above bars show number of morphospecies.

For the full dataset, the guild structure on four of the plant species (two within the Myrtaceae and two within the Proteaceae) was not significantly different between the control and warm site W1 (Table 2). There was less consistency in guild structure between the control site and warm site W2 with only one plant species (Myrtaceae) showing no significant difference. Guild structure between the two warm sites was largely consistent; there were no significant differences for six out of eight plant species (Table 2). For all eight plant species at all sites, the dominant guild was sapsuckers (phloem and mesophyll feeders combined) (Fig. 2). Differences in guild structure between the control site and the warm sites W1 and W2 were mainly driven by a reduction in sapsuckers and an increase in the predators and scavengers at the warm sites.

**Table 2:** Summary statistics for multivariate generalised linear model analyses (mGLM) of feeding guild species richness data, for the entire Coleoptera and Hemiptera guild structure.

Plant species	df a	Wald-*X^2^*	p			
			overall	C-W1	C-W2	W1-W2
Fabaceae						
*A. obtusata*	2,85	5.625	< 0.001	< 0.01	< 0.001	0.914
*A. parvipinnula*	2,87	8.17	< 0.001	< 0.01	< 0.001	< 0.001
*D. corymbosa*	2,87	4.49	< 0.001	< 0.05	< 0.01	< 0.05
Myrtaceae						
*A. hispida*	2,86	3.18	0.112	< 0.05	0.214	0.376
*C. pinifolius*	2,87	5.083	< 0.001	0.155	< 0.001	0.206
*L. squarrosum*	2,87	3.979	< 0.05	0.621	< 0.05	0.152
Proteaceae						
*H. gibbosa*	2,87	4.351	< 0.01	0.342	< 0.05	0.066
*T. speciosissima*	2,87	4.148	< 0.001	0.628	< 0.05	0.265

Pair wise comparisons between control and warm sites are shown.

a Sites: control (C), warm 1 (W1) and warm 2 (W2); degrees of freedom (df), Wald-*X^2^*-Statistic (Wald-*X^2^*) and p-value (p) overall and for pair wise comparisons between sites.

Within the herbivore dataset, feeding guild structure was largely consistent among sites (Fig. 2). There were no significant differences in guild structure between the control and warm site W1 for six of the eight species (two each within the Fabaceae, Myrtaceae and Proteaceae) (Table 3). The herbivore guild structure on two plant species (Myrtaceae) was not significantly different between the control and warm site W2. Guild structure between the two warm sites showed some consistency for four plant species, one from the Fabaceae and three from the Myrtaceae, with no significant differences between W1 and W2. For all eight plant species at all sites, the phloem feeders were the dominant herbivore guild (Fig. 2). Differences in herbivore guild structure between the control site and W1 were mainly caused by a reduction in phloem feeders (within the Fabaceae) or a reduction in leaf chewers (within the Myrtaceae) at warm site W1. At W2, differences in herbivore guild structure were mainly driven by either a reduction in phloem feeders at W2 (for three plant species, two Fabaceae and one Myrtaceae), or an increase in phloem feeders at W2 for three plant species (one Fabaceae and two Proteaceae). Differences in herbivore guild structure between the two warm sites were mainly due to an increase of mesophyll feeders at W1 for all four plant species (two each from Fabaceae and Myrtaceae).

**Table 3:** Summary statistics for multivariate generalised linear model analyses (mGLM) of feeding guild species richness data, for only the herbivore Coleoptera and Hemiptera guild structure; pair wise comparisons between sites are shown.

Plant species	df a	Wald-*X^2^*	p			
			overall	C-W1	C-W2	W1-W2
Fabaceae						
*A. obtusata*	2,85	5.51	< 0.001	< 0.001	< 0.001	0.575
*A. parvipinnula*	2,87	6.87	< 0.001	0.255	< 0.001	< 0.001
*D. corymbosa*	2,87	3.603	< 0.01	0.49	< 0.001	< 0.001
Myrtaceae						
*A. hispida*	2,86	2.996	0.07	< 0.05	0.061	0.121
*C. pinifolius*	2,87	4.512	< 0.001	0.14	< 0.001	0.068
*L. squarrosum*	2,87	2.284	0.478	0.402	0.744	0.363
Proteaceae						
*H. gibbosa*	2,87	3.967	< 0.01	0.462	< 0.01	< 0.01
*T. speciosissima*	2,87	3.638	< 0.01	0.092	< 0.001	< 0.05

a Sites: control (C), warm 1 (W1) and warm 2 (W2); degrees of freedom (df), Wald-*X^2^*-Statistic (Wald-*X^2^*) and p-value (p) overall and for pair wise comparisons between sites.

#### Comparison of guild distribution between transplants and congenerics at warm sites

For the full dataset, there was no consistency in community structure among the transplants at the two warm sites and congeneric plant species native to the warm area (Table 4, Fig. 2) for all except one of the five species-pair comparisons. Differences in guild structure between transplants at the warm sites and their congeneric partner plants were mainly driven by an increase of leaf chewers and predators at the congeneric species native to the warm area.

**Table 4:** Summary statistics for multivariate generalised linear model analyses (mGLM) of feeding guild species richness data, for the entire Coleoptera and Hemiptera guild structure; pair wise comparisons between the two warm sites and a congeneric host plant species in this area are shown.

Plant species	df a	Wald-*X^2^*	p		
			overall	W1-congener	W2-congener
Fabaceae					
*A. obtusata*	2,85	12.88	< 0.001	< 0.001	< 0.001
*A. parvipinnula*	2,87	7.79	< 0.001	< 0.01	< 0.001
Myrtaceae					
*C. pinifolius*	2,87	7.141	< 0.001	< 0.001	< 0.001
*L. squarrosum*	2,87	11.33	< 0.001	< 0.001	< 0.001
Proteaceae					
*H. gibbosa*	2,87	6.96	< 0.001	< 0.001	< 0.001

a Sites: warm 1 (W1) and warm 2 (W2), congeneric plant species (congener); degrees of freedom (df), Wald-*X^2^*-Statistic (Wald-*X^2^*) and p-value (p) overall and for pair wise comparisons between sites.

Within the herbivore dataset, there was no consistency in community structure between the transplants at the two warm sites and their congeneric partners (Fig. 2); the herbivore guild structure was significantly different for all five species-pair comparisons (Table 5). Differences in herbivore guild structure between transplants at the two warm sites W1 and W2 and their congeners were mainly due to a greater numbers of leaf chewers on the congeners (Fig. 2).

**Table 5:** Summary statistics for multivariate generalised linear model analyses (mGLM) of feeding guild species richness data, for only the herbivore Coleoptera and Hemiptera guild structure; pair wise comparisons between the two warm sites and a congeneric host plant species in this area are shown.

Plant species	df a	Wald-*X^2^*	p		
			overall	W1-congener	W2-congener
Fabaceae					
*A. obtusata*	2,85	10.72	< 0.001	< 0.001	< 0.001
*A. parvipinnula*	2,87	7.25	< 0.001	< 0.001	< 0.001
Myrtaceae					
*C. pinifolius*	2,87	6.004	< 0.001	< 0.001	< 0.01
*L. squarrosum*	2,87	8.133	< 0.001	< 0.001	< 0.001
Proteaceae					
*H. gibbosa*	2,87	5.001	< 0.001	< 0.05	< 0.001

a Sites: warm 1 (W1) and warm 2 (W2), congeneric plant species (congener); degrees of freedom (df), Wald-*X^2^*-Statistic (Wald-*X^2^*) and p-value (p) overall and for pair wise comparisons between sites.

#### General distribution of feeding guilds

For all transplanted plant species within the families Fabaceae and Myrtaceae, and all but one plant species within the Proteaceae, the dominant guild found at all sites within the entire Coleoptera and Hemiptera community was the sapsuckers (Fig. 2). Sapsuckers were also dominant on the congeneric plant species within the Fabaceae. Within the Myrtaceae, leaf chewers were also a major feeding guild. For one plant species within the Proteaceae (*H. gibbosa*), fungivores were the dominant guild at all sites, while for the congeneric plant species (*H. actites*), predators and leaf chewers were dominant (Fig. 2G). Within the herbivore subset, the dominant herbivores were phloem feeders for all transplanted plant species (Fig. 2), whereas for the congeneric species both leaf chewers and phloem feeders dominated.

## Discussion

The Coleoptera and Hemiptera fauna that colonised plants transplanted to warmer sites displayed an almost complete turnover of morphospecies composition compared to the control site within the native range after 12 months. This turnover could not be attributed to differences in plant growth between sites. The guild structure of the Coleoptera and Hemiptera communities was also markedly different between the warm and control sites for four of the eight host plant species. By contrast, when only the herbivores were considered, guild structure proved more consistent among sites, and this was reflected in a relatively consistent level of herbivore damage. These results suggest that as the climate warms, significant differences in the composition of insect communities could occur, but that at least within the herbivore component, species may tend to be progressively replaced by others within the same feeding guild.

### Community composition

There were marked differences in Coleoptera and Hemiptera morphospecies composition among all sites (see Table 1). Whilst a substantial component of this apparent turnover is likely to have been due, in part, to the under-sampling of the complete fauna, the results may also reflect a fundamental characteristic of the macroecology of Australian insects. Australian insects are generally considered to display a high level of endemism with narrow geographic ranges [74], [75]. A previous transplant experiment conducted in the same general region also found a high level of species turnover along the latitudinal extent of the host plant's range, as well as between the native and warmer transplant sites [53]. A transplant experiment, assessing impacts of climate warming on a montane meadow in Europe, also revealed that plant community composition was distinctly altered [45]. Similarly, a transplant experiment performed on soil nematode communities showed that community composition at the warmer sites was markedly altered [49]. A microarthropod community, subjected to experimentally increased temperature, also showed substantial changes in community composition [76]. Experimental temperature increases in an old-field experiment has also been found to alter insect community composition, particularly within the herbivore guilds [24]. Some of these studies attributed the changes in community composition to abiotic factors, such as global climate change drivers, [45], [49], [76] including an increase in temperature [24]. Several observational studies conducted along environmental temperature gradients, either latitudinal or altitudinal, have also found considerable turnover in insect community composition linked to climatic factors [37], [77]–[79].

We also found marked differences in Coleoptera and Hemiptera community composition between the transplants at the warm sites and their congeneric plant species in the warm area (see Table 1). This also indicates a high level of specialist species associated with Australia's flora [74], [75]. However, our findings contrast those of a previous study, in which threatened plant species were translocated in southwest Australia; insect assemblages were found to be similar to those of related plant species in the translocation area [80]. Again, our findings might be partly due to the under-sampling of the insect fauna.

We found that the morphospecies turnover between the two warm sites was very high, even though the two sites were only 8 km apart. This low overlap is likely to be at least partly due to insufficient sampling, a problem common to studies of insect diversity in which collection of a complete insect fauna is often prohibitive in terms of time and labour. Sampling is always a trade off between depth (e.g. taking many samples on one species, as in Andrew & Hughes [53]) and breadth (taking samples from many sites or many species). In this study we chose to focus on breadth so as to search for general patterns across multiple plant species.

Over the 12 month survey period, we found that there were generally more Coleoptera and Hemiptera morphospecies that colonised the transplants at the control site and W1 than at W2 (see Table S3 in file S1). This result may have been due to differences in habitat between the sites: the control site and W1 were both located in a dry sclerophyll forest with a tall eucalypt canopy that provided approximately 30–50% cover. W2, however, was located in coastal heath with sparse (∼20%) *Banksia* cover and had lower plant species diversity. This reduction in the local plant species richness and sparser structure may have led to a lower number of morphospecies at W2. Our findings are in accord with results from previous studies showing a positive relationship between Coleoptera and Hemiptera species richness and the structural complexity of vegetation [81], [82], or local plant species diversity [83], [84].

### Community structure – control vs. warm sites

The feeding guild structure of the Coleoptera and Hemiptera as a whole was relatively consistent between the control site and warm site W1; no significant differences were found in structure for four of the eight host plant species (two each from the Myrtaceae and Proteaceae) between these two sites (see Table 2). These consistencies were driven by similar distributions of particular feeding guilds, which were different within each family: Within the Proteaceae, numbers of members within all guilds were similar, and within the Myrtaceae minor feeding guilds (leaf chewer, scavenger and fungivore) were consistent (see Fig. 2). This suggests that individual host plant identity is an important driver of feeding guild structure.

We found that when differences in guild structure occurred, they were mainly driven by a reduction of sapsuckers and an increase of predators and scavengers at the warm sites (see Fig. 2). This suggests that predators and scavengers may benefit from warmer temperatures in the future. Similarly, increased temperature in an old-field experiment led to a distinct shift in guild structure, where numbers of predators significantly increased [24].

Coleoptera and Hemiptera feeding guild structure showed little consistency between the control site and W2, with seven of the eight plant species supporting significantly different proportions of feeding guilds. These differences were mainly driven by either higher numbers of fungivores (*D. corymbosa, A. hispida* and *C. pinifolius*) or fewer sapsuckers (*A. obtusata, A. parvipinnula, L. squarrosum, H. gibbosa* and *T. speciosissima*) at W2. The most likely factors contributing to these differences include the differences in structural complexity and plant species composition at W2. It has been shown previously that variations in structural complexity of sward, located at field margins in Great Britain, had a significant effect on the arthropod community structure [82], with phytophagous groups responding differently to an increase of structural complexity than predatory groups.

### Herbivore community structure – control vs. warm sites

The feeding guild structure of the herbivorous component of the Coleoptera and Hemiptera communities was quite consistent between the control site and warm site W1, with a significant difference between these two sites for two of the eight host plant species (see Table 3). These two sites had very similar vegetation and this result indicates that with a warming of 2–3°C little change in the broad structure of the phytophagous community might be expected (see also [53]). When comparisons were made between the control site and W2 however, little consistency in structure was found, with six of the eight plant species supporting significantly different proportions of guilds. As noted above, differences in the structure and species composition of the surrounding vegetation between these two sites are the most likely explanation for the differences, emphasising the potential importance of the ecological community context in determining assemblages on individual plant species.

### Congenerics vs. transplants at warm sites

There was a striking difference in the feeding guild structure between the transplants at the warm sites and their congeneric partner plant native to the warm sites (see Table 4, 5). This pattern was evident for both the entire Coleoptera and Hemiptera community and the phytophagous subset only. These differences were mainly driven by higher numbers of leaf chewers and predators on the congeneric partner plants, compared to the transplants. It is possible that differences in plant age contributed to these differences – the transplanted plants were all less than 2 years old whereas the congenerics were of mixed ages. However this result also indicates that the chemical and physical characteristics of individual plants species are important in driving the distribution of feeding niches on plants, and that there is significant variability in these traits, even within genera.

### Other approaches for studying climate change impacts

The results of this multi-species field transplant experiment complement findings from some earlier studies that employed different approaches to investigate the effects of increasing temperatures on plant-insect communities. These approaches include field-based warming experiments (e.g. [24], [41]), glasshouse experiments (e.g. [85], [86]), and species distribution models (e.g. [87], [88]). While such approaches are very useful when single species are considered, their usefulness for studying entire communities is limited. For example, field-based warming experiments are limited to the experimental plot; glasshouse experiments are isolated from the outside environment; and species distribution models, which are usually performed on single species only, cannot yet incorporate biotic interactions [89], [90]. Although field transplant experiments cannot incorporate future interactions (such as competition) between existing and colonising faunas, they offer useful tools for identifying broad impacts of future climate change on community structure and composition.

## Conclusion

We found an almost complete turnover in community composition between transplanted plants between their native range and warmer sites, indicating that with the current rate of warming a new suite of Coleoptera and Hemiptera species might colonise these eight host plant species within their native range during the next decades. Differences in feeding guild structure were also found, although at least some of these differences were likely to have been associated with differences in habitat types of the sites. We found more consistency in community structure of the herbivores, compared to the assemblage as a whole, suggesting that as phytophagous species migrate to track climate change they may colonise new host plants by replacing species within the same functional guild. While field transplant experiments such as this are time- and labour-intensive, they offer a valuable complement to laboratory and glasshouse experiments for understanding climate-change impacts.

## Supporting Information

File S1 Supportive information file containing feeding guild classification for Coleoptera and Hemiptera families (Table S1), Summary of ANOVA results for net growth rate of eight plant species after 12 months at all transplant sites (Table S2), Number of Coleoptera and Hemiptera morphospecies collected from each plant species at all sites (Table S3), and Coleoptera and Hemiptera community composition on Fabaceae (Fig. S1), Myrtaceae (Fig. S2) and Proteaceae species (Fig. S3).
(PDF)
