# Supplementary material for: Potential Impacts of Climate Change on Insect Communities: A Transplant Experiment
Source: PLoS One. 2014 Jan 22;9(1):e85987. doi: 10.1371/journal.pone.0085987 (PMC3899090; doi:10.1371/journal.pone.0085987)
Supplement: File S1 — Supportive information file containing feeding guild classification for Coleoptera and Hemiptera families (Table S1), Summary of ANOVA results for net growth rate of eight plant species after 12 months at all transplant sites (Table S2), Number of Coleoptera and Hemiptera morphospecies collected from each plant species at all sites (Table S3), and Coleoptera and Hemiptera community composition on Fabaceae (Fig. S1), Myrtaceae (Fig. S2) and Proteaceae species (Fig. S3). (PDF) [file pone.0085987.s001.pdf]

Supportive information

for

Potential impacts of climate change on insect communities: a transplant experiment

Sabine S. Nooten, Nigel R. Andrew, Lesley Hughes

Table S1: Feeding guild classification, based on mouthpart morphology and targeted plant tissue for (i) Coleoptera and (ii) Hemiptera families.

(i) Coleoptera

Feeding guild	Superfamily	Family
leaf chewer	Buprestoidea	Buprestidae
	Chrysomeloidea	Cerambycidae, Chrysomelidae
	Curculionidae	Attelabidae, Brentidae, Curculionidae
	Tenebrionoidea	Mordellidae, Scraptiidae
fungivore	Cleroidea	Phalacridae
	Cucujoidea	Corylophidae, Endomychidae, Lathridiidae, Mycetophagidae
	Elateroidea	Elateridae
	Staphylinoidea	Clambidae, Ptiliidae
	Tenebrionoidea	Myceteridae, Zopheridae
predator	Elateroidea	Cantharidae
	Caraboidea	Carabidae
	Cleroidea	Melyridae
	Cucujoidea	Coccinellidae, Bothrideridae
	Staphylinoidea	Pselaphidae, Staphylinidae
scavenger	Tenebrionoidea	Aderidae, Anthicidae, Oedemeridae, Tenebrionidae
	Bostrichoidea	Anobiidae, Dermestidae
	Elateroidea	Lycidae
	Cucujoidea	Nitidulidae
	Curculionoidea	Anthribidae
	Scarabaeoidea	Scarabaeidae

(ii) Hemiptera

Feeding guild	Suborder	Superfamily	Family/subfamily
phloem feeder [a]	Auchenorrhyncha	Fulgoroidea	Achilidae, Derbidae, Delphacidae, Eurybrachidae, Flatidae, Fulgoridae, Tropiduchidae
	Auchenorrhyncha	Membracoidea	Cicadellidae, subfamilies: Deltocephalinae, Eurymelinae, Iassinae, Tartessinae, Ulopinae, Xestocephalinae
		Membracoidea	Membracidae
	Sternorrhyncha	Psylloidea	Psyllidae
		Aphidoidea	Aphididae
		Aleyrodoidea	Aleyrodidae
		Coccoidea	Coccidae
mesophyll feeder [a]	Auchenorrhyncha	Membracoidea	Cicadellidae, subfamily Typhlocybinae
	Heteroptera	Lygaeoidea	Lygaeidae
		Miroidea	Miridae, Tingidae
		Pentatomoidea	Pentatomidae, Scutelleridae
xylem feeder [a]	Auchenorrhyncha	Cercopoidea	Cercopidae, Clastopteridae
predator	Heteroptera	Dipsocoroidea	Schizopteridae
		Lygaeoidea	Geocoridae
		Naboidea	Nabidae
		Reduvidoidea	Reduviidae
		Cimicoidea	Anthocoridae
seed predator	Heteroptera	Lygaeoidea	Rhyparochromidae, Cymidae
		Coreoidea	Alydidae

[a] mesophyll, phloem and xylem feeders are combined in a category of ‘sapsucker’ for the complete dataset but analysed separately for the herbivore dataset.

Table S2: Summary of ANOVA results and pairwise comparisons for net growth rate of eight plant species after 12 months at three transplant sites.

Plant species	df [a]	F	p			
			overall	C-W1	C-W2	W1-W2
Fabaceae						
A. obtusata	2,35	5.026	0.012	0.177	< 0.01	0.438
A. parvipinnula	2,67	0.370	0.692			
D. corymbosa	2,23	0.395	0.678			
Myrtaceae						
A. hispida	2,31	2.961	0.067			
C. pinifolius	2,76	10.241	< 0.001	0.01	0.264	< 0.001
L. squarrosum	2,65	2.068	0.135			
Proteaceae						
H. gibbosa	2,67	0.585	0.429			
T. speciosissima	2,28	30.068	< 0.01	< 0.01	< 0.01	0.985

[a] Sites: control (C), warm 1 (W1) and warm 2 (W2); degrees of freedom (df), F-Statistic (F) and p-value (p) overall and for pairwise comparisons between sites.

Table S3: Number of Coleoptera and Hemiptera morphospecies collected from each plant species at three sites, feeding guilds included. Superscript numbers (^{1,2}) show numbers of co-occurring morphospecies among sites and congeneric plant species (Conge), subscript ($_{1,2}$) among congeners and warm sites; Number of morphospecies collected from transplants at all three sites (# Msp).

Acacia obtusata

Family	Site			Conge	# Msp	Feeding guild
	C	W1	W2			
**Coleoptera**						
Aderidae				5		scavenger
Anobiidae		1		2	1	scavenger
Anthribidae			1	1	1	scavenger
Bothrideridae				1		predator
Brentidae	1			3	1	leaf chewer
Buprestidae			1_{1}	4_{1}	1	leaf chewer
Cantharidae				1		predator
Cerambycidae				2		leaf chewer
Chrysomelidae	1	3^{2}_{1}	4^{2}_{1}	7_{2}	6	leaf chewer
Coccinellidae	1	1	1	11	3	predator
Corylophidae		1^{1}	1^{1}	1	1	fungivore
Curculionidae	1		1_{1}	8_{1}	2	leaf chewer
Dermestidae		1				
Elateridae		1			1	fungivore
Endomychidae			1	1	1	fungivore
Lathridiidae	2^{2}	2^{2}		2^{1}	2	fungivore
Mordellidae				3		leaf chewer
Mycetophagidae				1		fungivore
Nitidulidae				1		scavenger
Oedemeridae		1			1	scavenger
Phalacridae		1^{1}	1^{1}	3^{1}	1	fungivore
Pselaphidae			1		1	predator
Ptiliidae	1^{1}	1^{1}	1^{1}	1^{1}	1	fungivore
Rhipiphoridae	1					parasite
Scarabaeidae		2^{1}_{1}	2^{1}_{1}	2_{1}	3	scavenger
Staphylinidae		2_{1}	1	3_{1}	3	predator
Tenebrionidae				1		scavenger
sum	*8*	*17*	*16*	*64*	*32*	
**Hemiptera**						
Achilidae		1		1	1	phloem feeder
Alydidae				2		seed predator
Aleyrodidae	1	1			2	phloem feeder
Anthocoridae				1		predator
Aphididae	6^{1}	2^{1}	5^{2}	5	11	phloem feeder
Cercopidae			1		1	xylem feeder
Coccidae	6^{1}	2^{1}	2	2^{1}	9	phloem feeder
Delphacidae		1	2	8	3	phloem feeder
Cicadellidae						
Deltocephalinae	2				2	phloem feeder
Iassinae		2		1	2	phloem feeder
Tartessinae			1	1	1	phloem feeder
Typhlocybinae	5	1^{1}	1^{1}	1^{1}	6	mesophyll feeder
Xestocephalinae	4		1_{1}	3_{1}	5	phloem feeder
Fulgoridae		1		1	1	phloem feeder

continued next page

Table S3: *continued*

**Family**	**Site**			**Conge**	**# Msp**	**Feeding guild**
	C	W1	W2			
Membracidae			1_1	3_1	1	phloem feeder
Miridae			1	5	1	mesophyll feeder
Nabidae	1	2^1			2	predator
Psyllidae	10	2_1	1	20_1	13	phloem feeder
Reduviidae				1^1		predator
Rhyparochromidae		1^1	1^1	2	1	seed predator
Tropiduchidae				3		phloem feeder
sum	*35*	*16*	*17*	*60*	*62*	

Acacia parvipinnula

**Family**	**Site**			**Conge**	**# Msp**	**Feeding guild**
	C	W1	W2			
**Coleoptera**						
Aderidae		3_1	1	5_1	4	scavenger
Anobiidae		1		2	1	scavenger
Anthribidae	2		1	1	3	scavenger
Bothrideridae				1		predator
Brentidae	1^1	5	1^1	3	5	leaf chewer
Buprestidae	1		1_1	4_1	2	leaf chewer
Cantharidae	1^1	1^1	1^1	1^1	1	predator
Carabidae	1				1	predator
Cerambycidae				2		leaf chewer
Chrysomelidae	8^3	8^2_1	11^3	7^1	20	leaf chewer
Clambidae			1		1	fungivore
Coccinellidae	1	9^2_3	4^2_2	11_4	12	predator
Corylophidae		1_1		1_1	1	fungivore
Curculionidae	1^1	5^1_1	3^2_1	8_1	7	leaf chewer
Endomychidae			1_1	1_1	1	fungivore
Lathridiidae	2^1	5^1	3^1	2^1	6	fungivore
Lycidae		1			1	scavenger
Melyridae		1		1	1	
Mordellidae		1	1_1	3_1	2	leaf chewer
Mycetophagidae				1		fungivore
Nitidulidae		1_1		1_1	1	scavenger
Phalacridae		1_1	1	3_1	2	fungivore
Ptiliidae		1^1	1^1	1^1	1	fungivore
Scarabaeidae	1	1_1	3_1	2_1	5	scavenger
Scirtidae		1			1	
Staphylinidae	1	6		3	7	predator
Tenebrionidae	1	1_1		1_1	2	scavenger
sum	*21*	*53*	*34*	*64*	*87*	
**Hemiptera**						
Achilidae				1		phloem feeder
Aleyrodidae	2	1			3	phloem feeder
Alydidae				2		seed predator
Anthocoridae			1	1	1	predator
Aphididae	5	4^1_1	4^1_2	5_2	12	phloem feeder
Blissidae		1			1	mesophyll feeder
Coccidae	4^1	2^2	4^2	2^1	7	phloem feeder
Cymidae			1		1	seed predator
Delphacidae	3	7_2	4	8_2	13	phloem feeder

continued next page

Table S3: *continued*

Family	Site			Conge	# Msp	Feeding guild
	C	W1	W2			
Cicadellidae						
Deltocephalinae	2	1^{1}	2^{1}		5	phloem feeder
Iassinae		3		1	3	phloem feeder
Tartessinae	1	1	2	1	4	phloem feeder
Typhlocybinae	8^{1}	10^{2}_{1}	6^{1}	1^{1}_{1}	22	mesophyll feeder
Xestocephalinae	2	4_{1}		3_{1}	6	phloem feeder
Eurybrachidae		1	1		1	phloem feeder
Fulgoridae		1_{1}		1_{1}	1	phloem feeder
Membracidae		4^{3}_{1}	4^{3}	3^{2}_{1}	5	phloem feeder
Miridae	1	1_{1}	3	5_{1}	5	mesophyll feeder
Nabidae	1	1	1		3	predator
Pentatomidae		1	2		3	mesophyll feeder
Psyllidae	15^{2}	11^{2}	1	20	25	phloem feeder
Reduviidae	2				2	predator
Rhyparochromidae			1_{1}	2_{1}	1	seed predator
Scutteleridae		1			1	mesophyll feeder
Tropiduchidae		1_{1}		3_{1}	1	phloem feeder
sum	*46*	*56*	*37*	*59*	*126*	

Daviesia corymbosa

Family	Site			# Msp	Feeding guild
	C	W1	W2		
**Coleoptera**					
Hydrophilidae		1		1	predator
Chrysomelidae		1		1	leaf chewer
Coccinellidae		2	1	3	predator
Curculionidae	2	1	1	4	leaf chewer
Dermestidae			1	1	scavenger
Endomychidae			1	1	fungivore
Mordellidae	1			1	leaf chewer
Nitidulidae		1		1	scavenger
Ptiliidae		1^{1}	1^{1}	1	fungivore
Scarabaeidae		1^{1}	1^{1}	1	scavenger
Staphylinidae		1		1	predator
Rhipiphoridae	1			1	parasite
sum	*4*	*9*	*6*	*17*	
**Hemiptera**					
Aphididae	5	5	4	16	phloem feeder
Coccidae	3^{1}	2^{1}		4	phloem feeder
Cicadellidae					
Deltocephalinae		2		2	phloem feeder
Tartessinae	1			1	phloem feeder
Typhlocybinae	5	6		11	mesophyll feeder
Xestocephalinae	1	2		3	phloem feeder
Delphacidae	2	4	2	8	phloem feeder
Fulgoridae		1		1	phloem feeder
Lygaeidae		1		1	mesophyll feeder
Nabidae		1	1	2	predator
Psyllidae	5	5		12	phloem feeder
Reduviidae	1	1		2	predator
Rhyparochromidae	1			1	seed predator
sum	*24*	*30*	*7*	*64*	

continued next page

Table S3: *continued*

Angophora hispida

Family	Site			# Msp	Feeding guild
	C	W1	W2		
**Coleoptera**					
Anobiidae		1		1	scavenger
Anthicidae		1	1	2	scavenger
Buprestidae	1			1	leaf chewer
Cantharidae		1		1	predator
Chrysomelidae	1		1	2	leaf chewer
Corylophidae		1		1	fungivore
Coccinellidae		1[1]	1[1]	2	predator
Curculionidae			1	1	leaf chewer
Mordellidae	1			1	leaf chewer
Phalacridae		1		1	fungivore
Pselaphidae		1		1	predator
Ptiliidae	1[1]	1[1]	1[1]	3	fungivore
Scarabaeidae		1		1	scavenger
Staphylinidae		2		1	predator
Tenebrionidae	1		1	2	scavenger
sum	*5*	*11*	*6*	*21*	
**Hemiptera**					
Aleyrodidae			2	2	phloem feeder
Aphididae	3	2	5	10	phloem feeder
Cercopidae		1		1	xylem feeder
Coccidae	1[1]	1	2[1]	3	predator
Delphacidae	2	4	1	7	phloem feeder
Cicadellidae					
Deltocephalinae		2	1	3	phloem feeder
Typhlocybinae	4	2[1]	1[1]	6	mesophyll feeder
Xestocephalinae	1			1	phloem feeder
Flatidae			1	1	phloem feeder
Lygaeidae			1	1	mesophyll feeder
Pentatomidae		1		1	mesophyll feeder
Psyllidae	5	1		6	phloem feeder
Schizopteridae			1	1	predator
Tropiduchidae			1	1	phloem feeder
sum	*16*	*14*	*16*	*44*	

Callistemon pinifolius

Family	Site			Conge	# Msp	Feeding guild
	C	W1	W2			
**Coleoptera**						
Anthribidae	1				1	scavenger
Brentidae	1			2	1	leaf chewer
Cantharidae		1		2	1	predator
Carabidae				1		predator
Chrysomelidae	1	2	2	2	5	leaf chewer
Corylophidae			1	2	1	fungivore
Coccinellidae		1		2_1	3	predator
Curculionidae	2			5	2	leaf chewer
Dermestidae			1		1	scavenger

continued next page

Table S3: *continued*

Family	Site			Conge	# Msp	Feeding guild
	C	W1	W2			
Elateridae				1		scavenger
Lathridiidae	1_1			2_1	1	fungivore
Mordellidae	1		1	1	2	leaf chewer
Phalacridae		1			1	fungivore
Pselaphidae			1		1	predator
Ptiliidae		1^1	2^1	1^1	2	fungivore
Scarabaeidae		1	1_1	1_1	3	scavenger
Staphylinidae		2_1	1	4_1	2	predator
sum	*7*	*9*	*10*	*26*	*27*	
**Hemiptera**						
Aleyrodidae		1		3	1	phloem feeder
Aphididae	8	5^2_1	3^2	2_1	14	phloem feeder
Cercopidae				1		xylem feeder
Delphacidae	1	1	2		4	phloem feeder
Cicadellidae						
Deltocephalinae		1			1	phloem feeder
Iassinae		2			2	phloem feeder
Typhlocybinae	4^1	4^1	3	1	10	mesophyll feeder
Xestocephalinae	3	1			4	phloem feeder
Coccidae	3	2	1		6	phloem feeder
Fulgoridae		1			1	phloem feeder
Lygaeidae			1		1	mesophyll feeder
Nabidae			1	1	1	predator
Pentatomidae			1	1	1	mesophyll feeder
Psyllidae	12	4		1	16	phloem feeder
Reduviidae				1		predator
Rhyparochromidae	2		1		3	seed predator
Schizopteridae				1		predator
sum	*33*	*22*	*13*	*12*	*65*	

Leptospermum squarrosum

Family	Site			Conge	#Msp	Feeding guild
	C	W1	W2			
**Coleoptera**						
Aderidae		1		1	1	scavenger
Anobiidae				2		scavenger
Anthribidae				1		scavenger
Attelabidae				2		leaf chewer
Brentidae		4_2		3_2	2	leaf chewer
Cantharidae		1_1		3_1	1	predator
Carabidae				1		predator
Chrysomelidae	2	2	2_1	3_1	6	leaf chewer
Clambidae		1			1	fungivore
Coccinellidae	1		1	8	2	predator
Colydiidae	1				1	fungivore
Corylophidae	1^1	2^1	1^1	1^1	1	fungivore
Curculionidae	2			11	4	leaf chewer
Dermestidae			1		1	scavenger
Elateridae				1		scavenger
Endomychidae		1		2	1	fungivore
Lathridiidae	2^1	2^1	1^1	2^1	3	fungivore
Melyridae	1			3	1	predator

continued next page

Table S3: *continued*

Family	Site			Conge	# Msp	Feeding guild
	C	W1	W2			
Mordellidae				1		leaf chewer
Myceteridae				2		fungivore
Nitidulidae		1			1	scavenger
Phalacridae				2		fungivore
Ptiliidae				1		fungivore
Scarabaeidae	2	1_{1}		3_{1}	3	scavenger
Scraptiidae				1		leaf chewer
Staphylinidae	1	2^{1}_{1}	1^{1}	4_{1}	3	predator
Tenebrionidae				1		scavenger
Zopheridae	1				1	fungivore
sum	*13*	*18*	*7*	*59*	*32*	
**Hemiptera**						
Aleyrodidae	1				1	phloem feeder
Aphididae	10^{1}	5^{2}	6^{2}	2	17	phloem feeder
Cicadellidae						
Deltocephalinae	5	1	3_{1}	3_{1}	9	phloem feeder
Eurymelinae				1		phloem feeder
Iassinae				1		phloem feeder
Ledrinae				1		phloem feeder
Typhlocybinae	9	3	3	6	15	mesophyll feeder
Xestocephalinae	2		1	2	3	phloem feeder
Coccidae	6	2		1	7	phloem feeder
Derbidae				1		phloem feeder
Delphacidae	1	5	4		10	phloem feeder
Eurybrachidae		1_{1}		1_{1}	1	phloem feeder
Flatidae		1		1	1	phloem feeder
Geocoridae				1		predator
Lygaeidae		1	2		3	mesophyll feeder
Miridae		1		1	1	mesophyll feeder
Machaerotidae	1				1	xylem feeder
Nabidae			1		1	predator
Pentatomidae				1		mesophyll feeder
Psyllidae	10	5_{1}		3_{1}	15	phloem feeder
Reduviidae			1		1	predator
Rhyparochromidae			1		1	seed predator
sum	*45*	*25*	*22*	*26*	*87*	

Hakea gibbosa

Family	Site			Conge	# Msp	Feeding guild
	C	W1	W2			
**Coleoptera**						
Aderidae				2		scavenger
Anthribidae				1		scavenger
Brentidae				4		leaf chewer
Buprestidae			1_{1}	1_{1}	1	leaf chewer
Cantharidae		1_{1}		2_{1}	1	predator
Chrysomelidae		3		4	3	leaf chewer
Corylophidae	1^{1}		1^{1}	3^{1}	1	fungivore
Coccinellidae	1		1		2	predator
Curculionidae	1			3	1	leaf chewer
Dermestidae			1		1	scavenger
Elateridae				1		scavenger

continued next page

Table S3: *continued*

Family	Site			Conge	# Msp	Feeding guild
	C	W1	W2			
Endomychidae		1[1]	1[1]		1	fungivore
Lathridiidae	2	2		1	4	fungivore
Melyridae		2		1	2	predator
Mordellidae		1		2	1	leaf chewer
Phalacridae		1			1	fungivore
Pselaphidae				5		predator
Ptiliidae	1[1]	1[1]	1[1]	1[1]	1	fungivore
Scarabaeidae		2		1	2	scavenger
Scraptiidae				1		leaf chewer
Scirtidae				1		leaf chewer
Staphylinidae		2	2_{1}	4_{1}	4	predator
Tenebrionidae			1	1	1	scavenger
sum	*6*	*16*	*9*	*39*	*27*	
**Hemiptera**						
Achilidae				3		phloem feeder
Aleyrodidae			1	1	1	phloem feeder
Aphididae	7	4[2]	3[2]	1[1]	12	phloem feeder
Cercopidae					1	xylem feeder
Cicadellidae						
Deltocephalinae		1		1	1	phloem feeder
Typhlocybinae	3[1]	2[2]	1[1]	2	4	mesophyll feeder
Xestocephalinae	4	1			5	phloem feeder
Tartessinae		1			1	phloem feeder
Coccidae	4[1]	1[1]		2	4	phloem feeder
Derbidae				1		phloem feeder
Delphacidae	3	6	1		10	phloem feeder
Lygaeidae			1		1	mesophyll feeder
Membracidae			1		1	phloem feeder
Miridae		1		2	1	mesophyll feeder
Psyllidae	6[3]	8[3]		6	11	phloem feeder
Reduviidae	1			3	1	predator
Rhyparochromidae	1				1	seed predator
Schizopteridae				1		predator
Tropiduchidae			1		1	phloem feeder
sum	*29*	*25*	*9*	*23*	*56*	

Telopea speciosissima

Family	Site			# Msp	Feeding guild
	C	W1	W2		
**Coleoptera**					
Anthribidae			1	1	scavenger
Attelabidae			1	1	leaf chewer
Cantharidae		1		1	predator
Chrysomelidae	2		1	3	leaf chewer
Curculionidae		1		1	leaf chewer
Corylophidae	1[1]	1[1]		1	fungivore
Dermestidae		1		1	scavenger
Endomychidae			1	1	fungivore
Lathridiidae	1		1	2	fungivore
Melyridae	1			1	predator
Mordellidae	1			1	leaf chewer

continued next page

Table S3: *continued*

**Family**	**Site**			**Conge**	**# Msp**	**Feeding guild**
	C	W1	W2			
Ptiliidae	1[1]	1[1]	1[1]	1		fungivore
Scarabaeidae		1		1		scavenger
Staphylinidae		1	1	2		predator
sum	*7*	*7*	*7*	*18*		
**Hemiptera**						
Aphididae	4	4[1]	4[1]	11		phloem feeder
Delphacidae	1	2		3		phloem feeder
Cicadellidae						
Deltocephalinae	1			1		phloem feeder
Iassinae		1		1		phloem feeder
Tartessinae		1		1		phloem feeder
Typhlocybinae	4	2		6		mesophyll feeder
Xestocephalinae		1		1		phloem feeder
Coccidae	4	1		5		phloem feeder
Fulgoridae		1		1		phloem feeder
Membracidae		1		1		phloem feeder
Miridae	1			1		mesophyll feeder
Psyllidae	4	3	2	9		phloem feeder
Tropiduchidae	1			1		phloem feeder
sum	*20*	*17*	*6*	*42*		

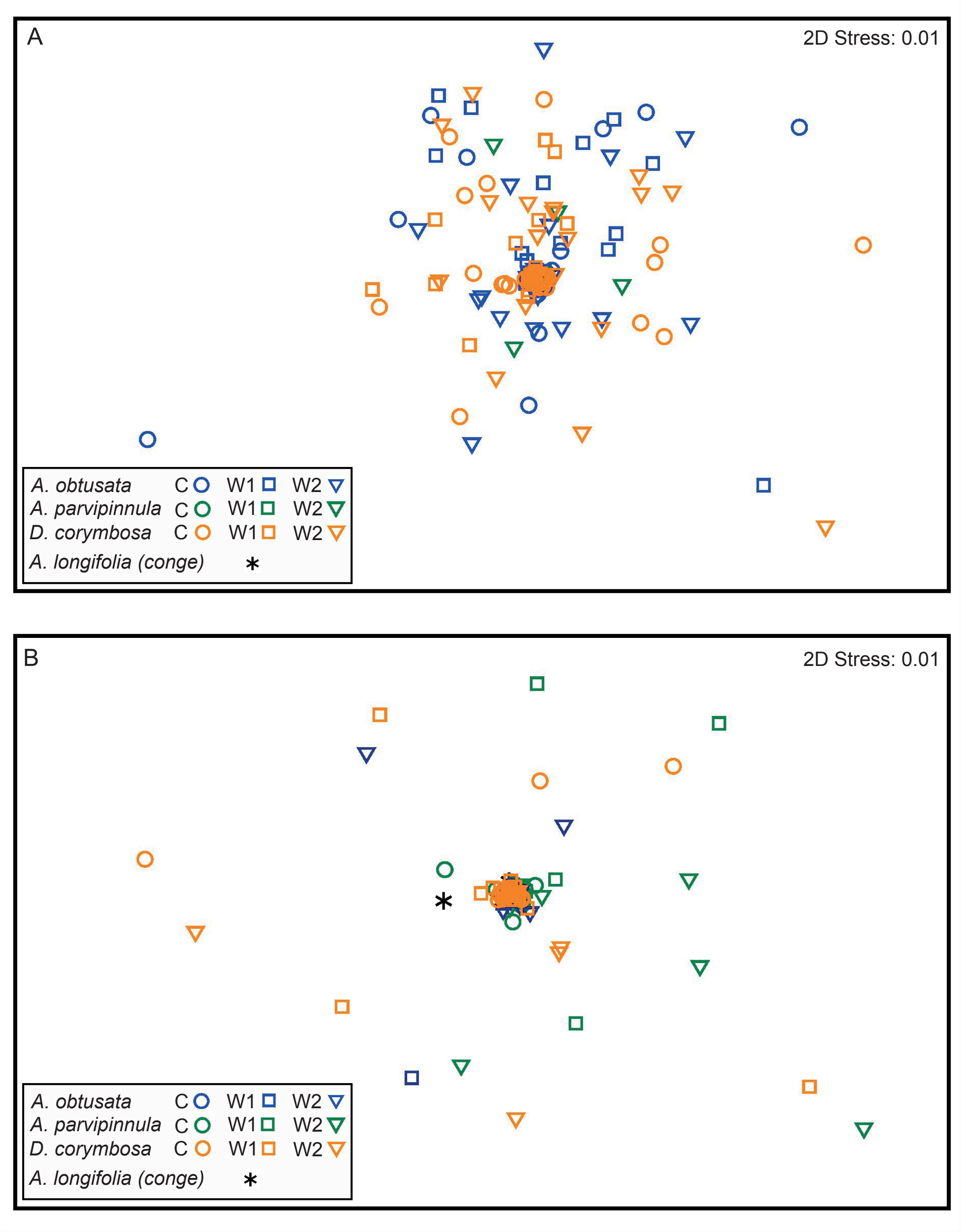

Figure S1: Community composition for Fabaceae species at all transplant sites. nMDS plots show community composition for morphospecies from (A) the full dataset and (B) the herbivore dataset, for three transplanted plant species and one congeneric plant species (conge).

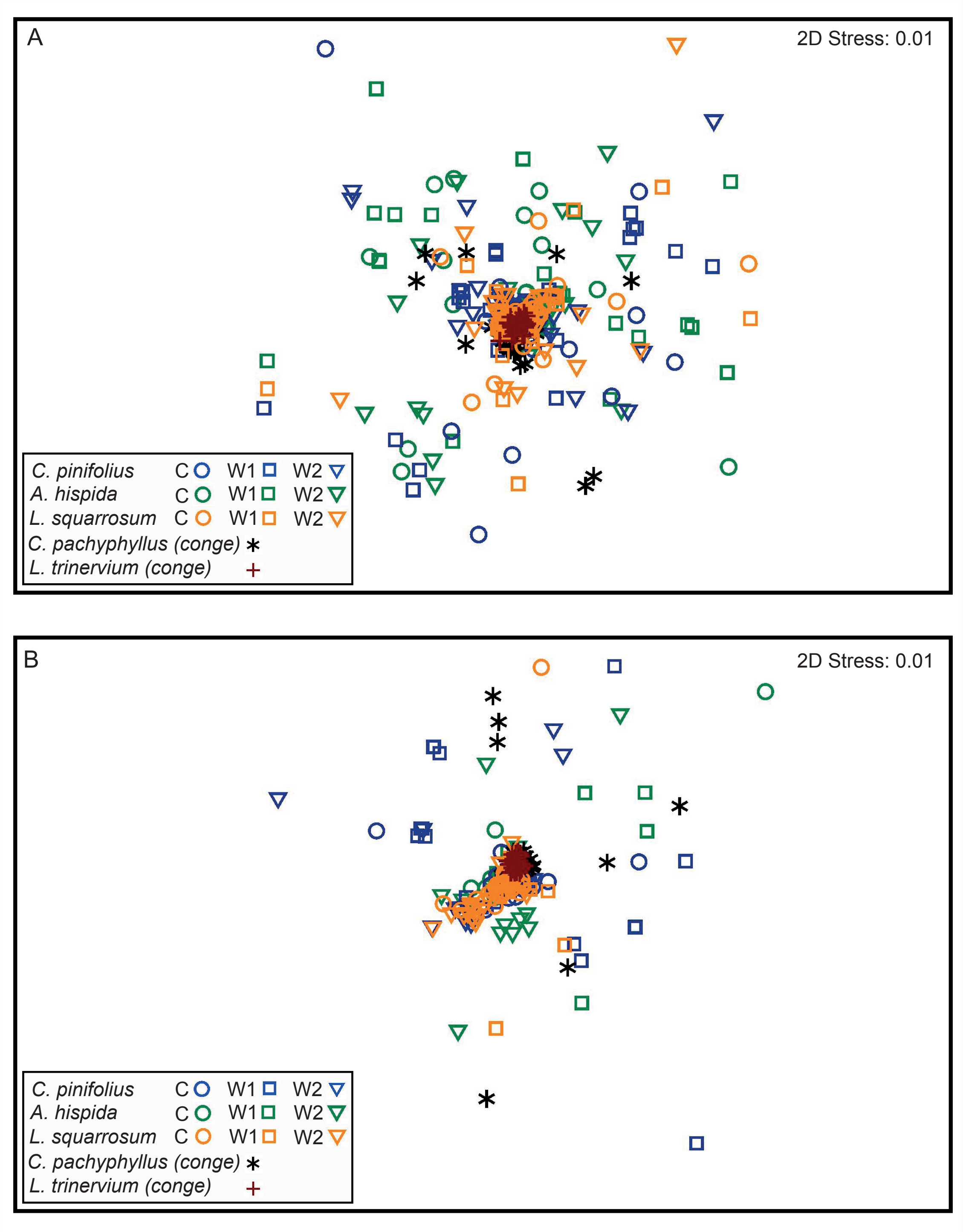

Figure S2: Community composition for Myrtaceae species at all transplant sites. nMDS plots show community composition for morphospecies from (A) the full dataset and (B) the herbivore dataset, for three transplanted plant species and two congeneric plant species (conge).

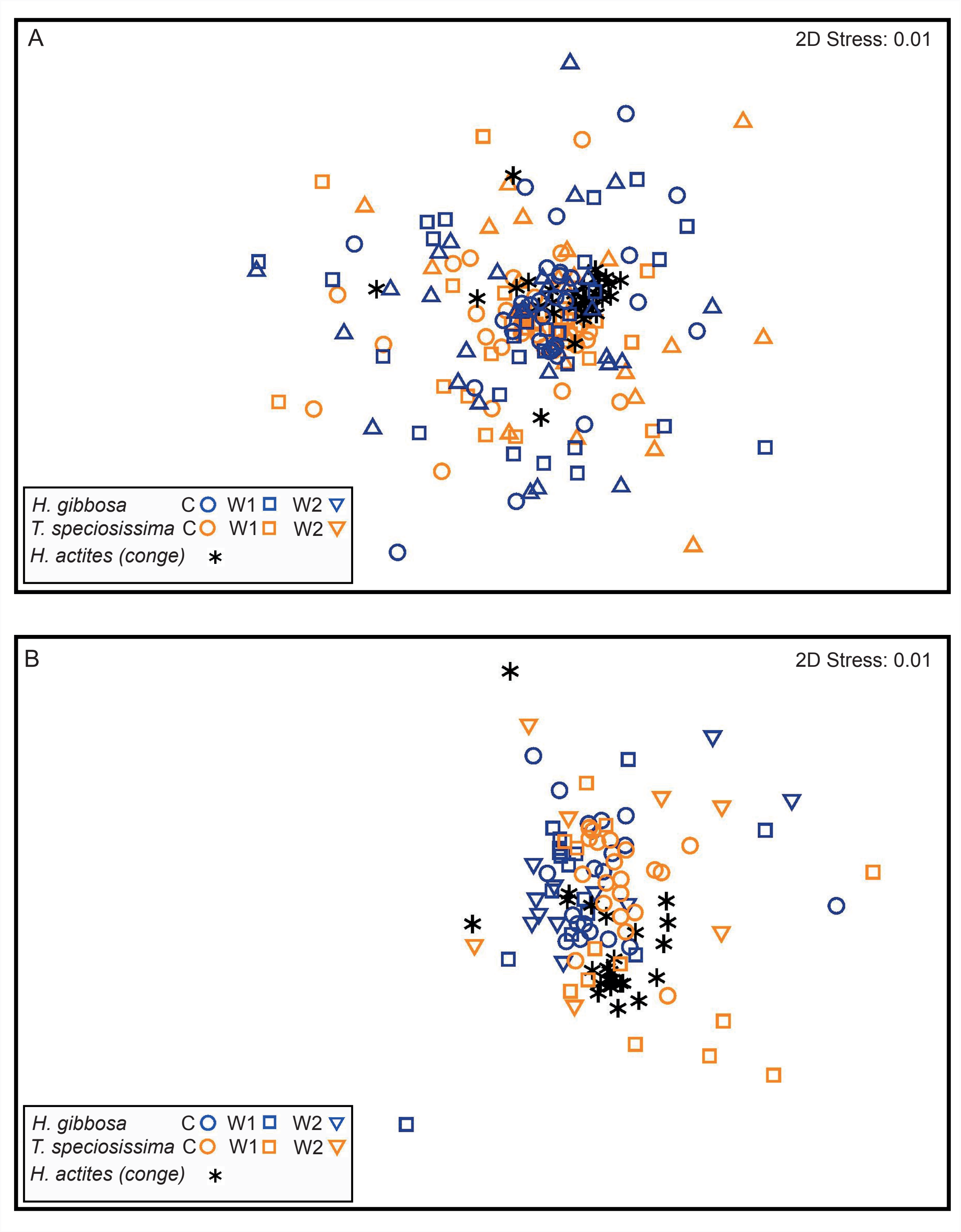

Figure S3: Community composition for Proteaceae species at all transplant sites. nMDS plots show community composition for morphospecies from (A) the full dataset and (B) the herbivore dataset, for two transplanted plant species and one congeneric plant species (conge).
